# Association between different composite dietary antioxidant indexes and low back pain in American women adults: a cross-sectional study from NHANES

**DOI:** 10.1186/s12889-024-17649-0

**Published:** 2024-01-10

**Authors:** Chaoqun Feng, Junjie Yao, Yizhou Xie, Fei Yang, Xiaohong Fan

**Affiliations:** 1 Department of Orthopedics, Hospital of Chengdu University of Traditional Chinese Medicine, 610075 Chengdu, P. R. China; 2 College of Acupuncture and Tuina, Changchun University of Chinese Medicine, 130117 Changchun, Jilin P.R. China

**Keywords:** Antioxidant, Composite dietary antioxidant index (CDAI), Low back pain, National Health and Nutrition Examination Survey ( NHANES), Oxidative stress

## Abstract

**Background:**

Low back pain is the leading cause of productivity loss, imposes a significant economic burden on the patients and society. Oxidative stress is considered a critical factor in the complex pathophysiological process and pathogenic mechanism of low back pain. Adjustment dietary pattern can effectively increase antioxidant biomarkers levels within the body to reduce oxidative stress. The composite dietary antioxidant index (CDAI) serves a reliable scoring system for quantifying the potential dietary antioxidant capacity of daily diets.

**Objective:**

We aim to investigate the potential association between CDAI and low back pain, in order to enhance the management of low back pain through dietary guidance.

**Methods:**

This study included 17,682 participants from the National Health and Nutrition Examination Survey (NHANES) 1999–2000, 2001–2002, 2003–2004 and 2009–2010. The weighted logistic regression model was used to investigate the association between CDAI and low back pain, while restricted cubic spline (RCS) was employed to examine non-linear trend and cutoffs.

**Results:**

After adjusting for all confounders, the results showed that there was no significant association between CDAI and low back pain. However, individuals in the highest quartile of CDAI exhibited an 11.7% less likelihood of experiencing a low back pain than those in the lowest quartile (OR = 0.883; 95% CI [0.787,0.991], *P* = 0.034), and the trend test was also significant (P for trend < 0.001). RCS indicated a linear relationship between CDAI and low back pain (P for non-linear = 0.876). Gender subgroup analysis showed that this negative association was significant in the female population (OR = 0.983; 95% CI [0.968, 0.998], *P* = 0.027), and females in the highest quartile of CDAI were 19.7% less likely to suffer low back pain than those in the lowest quartile (OR = 0.803; 95% CI [0.682,0.945], *P* = 0.008). Additionally, the changes in zinc (OR = 1.009; 95% CI [1.002, 1.016], *P* = 0.015) and selenium (OR = 0.379; 95% CI [0.164, 0.875], *P* = 0.023) per milligram were independently associated with low back pain.

**Conclusion:**

The fully adjusted model showed no significant association between CDAI and low back pain, but it was significant in quartiles. Meanwhile, subgroup analysis by gender revealed a negative association between CDAI and low back pain in the female population. Additionally, the findings of this study also suggested that the antioxidant diets should be studied in a dietary pattern context.

**Supplementary Information:**

The online version contains supplementary material available at 10.1186/s12889-024-17649-0.

## Introduction

Low back pain covers a spectrum of different types of pain, including nociceptive pain, neuropathic (radicular) pain, nociplastic pain, and non-specific pain [1]. It is normally considered as pain, muscle tension or stiffness localized below the costal margin and above the inferior gluteal folds, with or without sciatica [2]. Worldwide, approximately 37% of adults suffers from low back pain, which places a huge economic burden on the individuals and society [3]. According to the 2017 Global Burden of Disease Study findings, low back pain ranked first in terms of productivity loss measured in years, and was the top cause of years lived with disability in 126 countries [4]. Factors associated with low back pain include ageing, obesity, physical inactivity, lifestyle factors, depression and other psychosocial aspects [5]. Despite these challenges, steady progress has been achieved in the understanding of low back pain. These recent findings have contributed to the development of new diagnostic procedures and more targeted interventions [2].

Oxidative stress is considered a critical factor in the complex pathophysiological process and pathogenic mechanism of low back pain [6]. The delicate balance between reactive oxygen species (ROS) and antioxidants is essential for maintaining normal function and tissue structure. Current studies have shown that oxidative stress can promote the progression of low back pain through multiple pathways [7, 8], and inhibiting excessive ROS production while promoting its clearance has been proven effective in delaying intervertebral disc degeneration [9, 10]. The pathological process and pathogenic mechanism also provide potential therapeutic strategies.

Diet plays a crucial role in providing exogenous antioxidants, which effectively increase the levels of antioxidant biomarkers in the body to reduce oxidative stress [11]. Adjusting dietary patterns may be an effective approach to alleviate low back pain. The composite dietary antioxidant index (CDAI) serves as a reliable scoring system for quantifying the potential dietary antioxidant capacity of daily diet [12]. Previous studies have found that CDAI is inversely related to the prevalence of osteopenia [13], hypertension [14], depression [15] and cardiovascular mortality [16]. However, other studies have shown that the dietary antioxidant capacity is directly proportional to obesity, which can lead to cardiovascular disease, type 2 diabetes, and cancers [17]. Additionally, there is significant dimorphism in low back pain and oxidant balance based on gender differences [1, 18]. Currently, the relationship between CDAI and low back pain has not been evaluated. Our intention is to investigate the potential link between CDAI and low back pain, with the aim of better managing it through dietary guidance.

## Materials and methods

### Study population

The study involved participants from the National Health and Nutrition Examination Survey (NHANES), which combines information from interviews, physical examinations and various laboratory tests to assess the health and nutritional status of adults and children in the United States. Due to the limited data availability on low back pain in subsequent years, only datasets for four periods were used to analyze the association between CDAI and low back pain. Therefore, further details were collected from NHANES 1999–2000, 2001–2002, 2003–2004 and 2009–2010. Individuals with missing dietary data and low back pain data were excluded from the interested datasets. The survey protocols received approval from the Ethics Review Board of the National Center for Health Statistics (NCHS), and documented consent was obtained from participants (Protocol #98 − 12, Continuation of Protocol #2005-06).

**Fig. 1:**
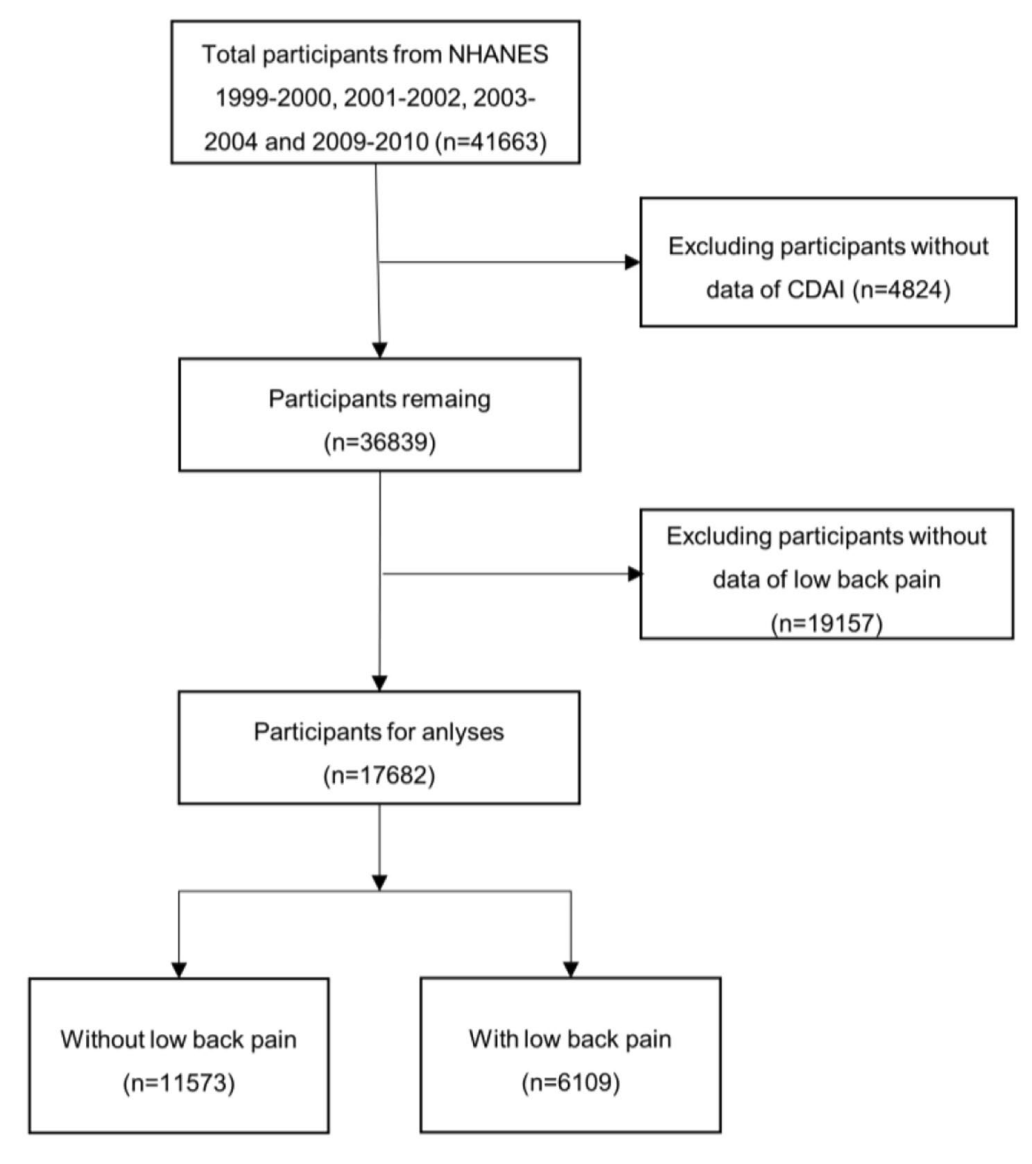
The flow diagram of participants selection

The initial search identified 41,663 participants for consideration from the NHANES 1999–2004 and 2009–2010. After excluding 4824 individuals without dietary data and 19,157 without low back pain data, a total of 17,682 adults were eventually included into our study. The flow diagram for participants selection is detailed in Fig. 1.

### Exposure and outcomes

The dietary intake data of all participants were recorded through 24-hour dietary recall interviews from midnight to midnight. The Food and Nutrient Database for Dietary Studies of the United States Department of Agriculture (USDA) was used to investigate the intakes of antioxidants [19]. For the datasets that release two days data, the average values of antioxidants were used for subsequent calculations. Based on the questionnaire interview, we determined the antioxidant components, including vitamins A, C, E, carotenes, zinc, and selenium. Additionally, the NHANES 1999–2000 antioxidant components were calculated using an earlier version of the database known as USDA 1994–1998 Survey Nutrient Database. Fortunately, the CDAI calculation could avoid the problem that some antioxidant units are inconsistent with other dietary data of different periods. Six antioxidants were standardized by subtracting the mean and dividing by the standard deviation. Then the CDAI was calculated based on the sum of these standardized values.

$$ CDAI={\sum }_{n=1}^{6}\left( \frac{{x}_{n}-{\mu }_{n}}{{s}_{n}}\right)$$

In this formula, $$ {x}_{n}$$ represents the daily intake of antioxidant components; $$ {\mu }_{n}$$ represents the mean of $$ {x}_{n}$$; $$ {s}_{n}$$ represents the standard deviation for $$ {\mu }_{n}$$ [20].

The NHANES collected pain data from participants aged 20 and older. Low back pain as the main outcome variable was only tested in the four NHANES cycles of 1999–2000, 2001–2002, 2003–2004 and 2009–2010. In this study, low back pain was classified as a binary outcome based on the response to the question “During the last 3 months, did you have low back pain?”

### Covariates

In order to eliminate the influence of potential confounding factors, several closely related covariates were selected, including gender, age, race, education, family poverty income ratio (PIR), body mass index (BMI), activity condition, and smoking [5]. The covariates were collected by questionnaires and physical examinations. The missing values in the data acquisition of various covariates were classified into a separate group. Family PIR was categorized into three levels: low income (< 1.3), medium income (1.3–3.5), and high income (≥ 3.5). BMI was calculated using height and weight, with the formula being weight divided by the square of height in kg/m^2^. We used standard WHO criteria to define underweight (< 18.5), normal (18.5–25), overweight (25–30) and obesity (≥ 30) [21]. The physical activity questionnaire was used to evaluate activity condition. Based on the suggested metabolic equivalent (MET) scores by NHANES, the activity condition was classified into inactive, moderate, and vigorous. Smoking was defined by a “yes” response to the question “Have you smoked at least 100 cigarettes in your entire life?”

### Statistical analysis

As recommended by the NCHS, the MEC exam weight were incorporated into our analyses to account for the complex study design. NHANES data from 1999 to 2002 were comprehensively analyzed using a set of four-year sample weight, while 2003–2004 and 2009–2010 using two-year sample weight. Participants were separated into four groups based on the quartile of CDAI values. Continuous variables were presented as means with standard errors, while categorical variables were expressed as percentages and 95% confidence intervals (CIs). The association between baseline characteristics and quartiles of CDAI was assessed using chi-square tests or t-tests. The weighted logistic regression model was used to explore the association between CDAI and low back pain, and the results were presented in the form of adjusted odds ratios (ORs) and 95% CIs. Model 1 was adjusted for none, and model 2 was adjusted for gender, age and race. Model 3 added education level, family PIR, BMI, activity condition and smoking as covariates to model 2. The variance inflation factor was calculated to evaluate the multicollinearity of covariates. The medians of CDAI quartiles were regarded as a continuous variable to explore the linear trend. Using the “rcssci” R package [22, 23], we added a restricted cubic spline (RCS) term on CDAI in the weighted logistic regression adjusted on confounding factors according to Model 3. The number of knots was chosen between 3 and 7 by minimizing the AIC. R software (version 4.2.0) and Stata (version 17.0) were used for all analyses (See Supplementary material 1), and *p* value less than 0.05 was considered statistically significant.

## Results

### Demographics

This study included a total of 17,682 eligible participants for analysis. Table 1 showed the baseline characteristics of the study population according to CDAI quartiles, revealing significant differences in age, gender, race, education level, family PIR, BMI, activity condition and smoking among different quartiles (*P* < 0.001). In contrast, the highest CDAI quartile group tended to be younger, male, non-Hispanic white, higher education, better economy, 18.5 to 30 BMI, vigorous activity, and non-smoking. Additionally, a significant association between CDAI in quartile and low back pain was found in the absence of covariates (*p* < 0.001).

**Table 1:** The baseline characteristics of the study population by CDAI quartiles

Variable	Total (*n* = 17,682)	Quartiles of composite dietary antioxidant index (CDAI)				*P*
		Q1 (*n* = 4421) <-2.219	Q2 (*n* = 4420) -2.219 ~ -0.259	Q3 (*n* = 4420) -0.259 ~ 2.353	Q4 (*n* = 4421) ≥ 2.353	
Age [mean (SEM)]	45.16 ± 0.13	46.20 ± 0.30	45.41 ± 0.28	45.71 ± 0.26	43.55 ± 0.24	< 0.001
Gender [N (%)]						< 0.001
Male	48.42(47.50,49.35)	32.41(30.69,34.18)	41.09(39.26,42.93)	51.42(49.59,53.26)	65.4(63.67,67.09)	
Female	51.58(50.65,52.50)	67.59(65.82,69.31)	58.91(57.07,60.74)	48.58(46.74,50.41)	34.6(32.91,36.33)	
Race [N (%)]						< 0.001
Mexican American	7.84(7.54,8.15)	8.39(7.76,9.07)	8.04(7.45,8.67)	7.45(6.87,8.07)	7.58(7.00,8.20)	
Non-Hispanic White	70.18(69.43,70.92)	64.63(62.97,66.26)	69.75(68.20,71.26)	71.78(70.30,73.21)	73.60(72.21,74.94)	
Non-Hispanic Black	11.08(10.67,11.49)	15.45(14.46,16.49)	10.68(9.90,11.53)	9.44(8.72,10.21)	9.41(8.71,10.15)	
Other Race	10.90(10.34,11.50)	11.53(10.34,12.84)	11.52(10.36,12.80)	11.33(10.22,12.55)	9.42(8.43,10.51)	
Education level [N (%)]						< 0.001
<High School	44.80(43.89,45.71)	56.36(54.45,58.25)	47.52(45.67,49.38)	40.39(38.64,42.17)	37.01(35.33,38.73)	
≥High School	55.06(54.14,55.97)	43.58(41.68,45.49)	52.23(50.37,54.08)	59.50(57.72,61.26)	62.83(61.11,64.51)	
Unknow	0.15(0.09,0.23)	0.06(0.02,0.17)	0.25(0.11,0.54)	0.10(0.05,0.24)	0.16(0.06,0.43)	
Family PIR [N (%)]						< 0.001
< 1.3	19.87(19.21,20.56)	27.31(25.76,28.91)	21.55(20.14,23.03)	16.86(15.67,18.11)	15.12(14.02,16.29)	
1.3–3.5	33.42(32.56,34.29)	36.38(34.58,38.21)	33.86(32.14,35.62)	32.64(30.95,34.37)	31.33(29.70,33.01)	
≥ 3.5	39.31(38.39,40.25)	29.02(27.22,30.89)	36.32(34.48,38.19)	43.47(41.63,45.33)	46.53(44.72,48.35)	
Unknow	7.40(6.95,7.87)	7.29(6.43,8.26)	8.28(7.34,9.33)	7.03(6.20,7.97)	7.02(6.19,7.96)	
BMI [kg/m^2^, N (%)]						< 0.001
underweight	1.57(1.35,1.82)	1.98(1.5,2.6)	1.46(1.05,2.03)	1.44(1.07,1.94)	1.45(1.08,1.95)	
normal	34.26(33.38,35.15)	35.63(33.81,37.48)	33.66(31.89,35.48)	33.42(31.71,35.18)	34.49(32.79,36.23)	
overweight	33.87(33.00,34.75)	30.81(29.11,32.57)	34.87(33.11,36.67)	34.38(32.65,36.15)	34.99(33.29,36.73)	
obesity	28.51(27.69,29.35)	29.09(27.41,30.84)	27.96(26.36,29.63)	29.37(27.73,31.06)	27.71(26.11,29.36)	
Unknow	1.79(1.60,2.00)	2.49(2.07,2.99)	2.04(1.63,2.55)	1.39(1.09,1.79)	1.36(1.04,1.77)	
Activity scores [N (%)]						< 0.001
Inactive	23.89(23.15,24.65)	30.82(29.16,32.53)	25.54(24.03,27.10)	22.07(20.66,23.54)	18.45(17.16,19.81)	
Moderate	37.28(36.38,38.18)	39.51(37.65,41.39)	38.28(36.47,40.12)	37.81(36.04,39.62)	34.03(32.33,35.77)	
Vigorous	38.83(37.92,39.74)	29.67(27.95,31.46)	36.19(34.38,38.03)	40.12(38.32,41.95)	47.52(45.72,49.33)	
Smoking [N (%)]						< 0.001
Yes	48.33(47.41,49.26)	52.53(50.65,54.42)	48.28(46.41,50.15)	46.44(44.62,48.28)	46.74(44.94,48.54)	
No	51.59(50.67,52.52)	47.39(45.51,49.28)	51.72(49.85,53.59)	53.41(51.57,55.23)	53.19(51.39,54.99)	
Unknow	0.07(0.04,0.14)	0.07(0.02,0.25)	NA	0.15(0.06,0.36)	0.07(0.01,0.33)	
Low back pain [N (%)]						< 0.001
Yes	35.38(34.49,36.27)	38.48(36.65,40.34)	34.92(33.16,36.72)	35.20(33.46,36.98)	33.41(31.72,35.15)	
No	64.62(63.73,65.51)	61.52(59.66,63.35)	65.08(63.28,66.84)	64.80(63.02,66.54)	66.59(64.85,68.28)	

Mean ± SD for continuous variables: the *P* value was calculated by the weighted linear regression model (%) for categorical variables: the *P* value was calculated by the weighted chi-square test

Abbreviations: *BMI* body mass index; *Family PIR* family poverty income ratio; *CDAI* composite dietary antioxidant index

### The association between CDAI and low back pain

**Fig. 2:**
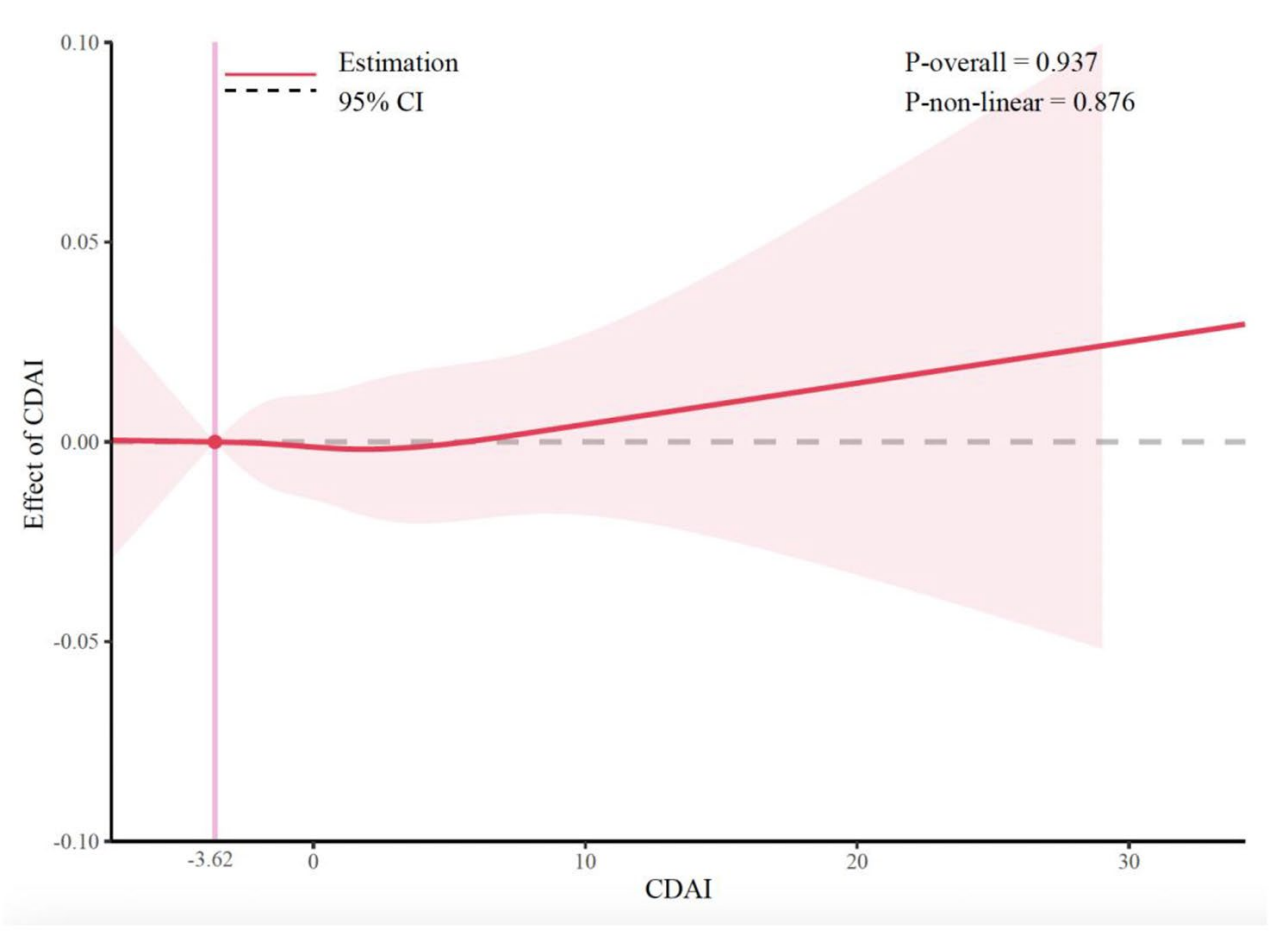
The dose–response relationships of CDAI with the prevalence of low back pain. The solid red line represents the smooth curve fit between variables. The shaded bands represent the 95% confidence intervals. The intersection of the pink line is the cutoff point

Table 2 showed the logistic regression weighted model of CDAI and low back pain. In the Model 1 and Model 2, CDAI was significantly negative correlated with low back pain (OR = 0.984; 95% CI [0.975, 0.993], *P* < 0.001; OR = 0.987; 95% CI [0.978, 0.997], *P* = 0.008), which indicated that the prevalence of low back pain was reduced for each additional unit rise in CDAI. However, this association disappeared in the Model 3 with increased covariates (OR = 0.993; 95% CI [0.983, 1.002], *P* = 0.138). We calculated that the variance inflation factor of each covariable was less than 5 in Model 3, indicating that there was no multicollinearity among them (See Supplementary material 2: Table S1). After transforming the CDAI into quartiles, we found that individuals with the highest quartile of CDAI were 11.7% less likely to have low back pain than those with the lowest quartile (OR = 0.883; 95% CI [0.787,0.991], *P* = 0.034), and the trend test was also significant (P for trend < 0.001). This negative relationship remained stable in the second quartile of CDAI (OR = 0.881; 95% CI [0.787, 0.986], *P* = 0.027). According to the minimum principle of AIC, RCS were used with four knots at the 5th, 35th, 65th, and 95th centiles to flexibly model the association between CDAI and low back pain. As shown in Fig. 2, RCS indicated that the relationship between CDAI and low back pain was linear (P for non-linear = 0.876). When the knots were3, 5, 6, and 7 respectively, this relationship also remained linear (See Supplementary material 2: Figure S1).

**Table 2:** The weighted logistic regression analysis of the association between CDAI and low back pain

Exposures	Model 1		Model 2		Model 3	
	OR [95%CI]	*P*	OR [95%CI]	*P*	OR [95%CI]	*P*
CDAI	0.984(0.975,0.993)	**< 0.001**	0.987(0.978,0.997)	**0.008**	0.993(0.983,1.002)	0.138
Quintiles of CDAI						
Q1 (<-2.219)	Ref	-	Ref	-	Ref	-
Q2 (-2.219 ~ -0.259)	0.858(0.768,0.958)	**0.007**	0.859(0.769,0.961)	**0.008**	0.881(0.787,0.986)	**0.027**
Q3 (-0.259 ~ 2.353)	0.869(0.778,0.970)	**0.012**	0.876(0.784,0.980)	**0.020**	0.916 (0.818,1.030)	0.126
Q4 (≥ 2.353)	0.802(0.719,0.895)	**< 0.001**	0.829(0.740,0.928)	**0.001**	0.883(0.787,0.991)	**0.034**
*P* for trend	**< 0.001**		**< 0.001**		**< 0.001**	

Model 1: no covariates were adjusted

Model 2: adjusted for gender, age, and race

Model 3: adjusted for gender, age, race, education level, family PIR, BMI, activity condition, and smoking

Abbreviations: *BMI* body mass index; *Family PIR* family poverty income ratio; *CDAI* composite dietary antioxidant index

As shown in Table 3, we conducted a stratified analysis based on gender difference. A significant negative association was found between CDAI and low back pain in female patients (OR = 0.983; 95% CI [0.968, 0.998], *P* = 0.027). Furthermore, females in the highest quartile of CDAI were 19.7% lower likely to experience low back pain compared to those in the lowest quartile (OR = 0.803; 95% CI [0.682,0.945], *P* = 0.008).

**Table 3:** The weighted logistic regression analysis of the association between CDAI and low back pain in different genders

Exposure	Model 1		Model 2		Model 3	
	OR [95%CI]	*P*	OR [95%CI]	*P*	OR [95%CI]	*P*
Male						
Q1 (<-2.219)	Ref	-	Ref	-	Ref	-
Q2 (-2.219 ~ -0.259)	0.901(0.749,1.084)	0.270	0.872(0.724,1.050)	0.148	0.902(0.748,1.088)	0.279
Q3 (-0.259 ~ 2.353)	0.971(0.815,1.156)	0.741	0.936(0.785,1.116)	0.461	0.966(0.808,1.154)	0.703
Q4 (≥ 2.353)	0.941(0.796,1.112)	0.477	0.908(0.766,1.076)	0.263	0.964(0.811,1.145)	0.676
CDAI	0.996(0.984,1.008)	0.512	0.995(0.983,1.007)	0.395	0.999(0.987,1.011)	0.863
Female						
Q1 (<-2.219)	Ref	-	Ref	-	Ref	-
Q2 (-2.219 ~ -0.259)	0.859(0.748,0.988)	**0.033**	0.860(0.748,0.989)	**0.035**	0.878(0.762,1.011)	0.071
Q3 (-0.259 ~ 2.353)	0.851(0.735,0.984)	**0.030**	0.845(0.730,0.978)	**0.024**	0.887(0.765,1.030)	0.115
Q4 (≥ 2.353)	0.751(0.640,0.881)	**< 0.001**	0.751(0.639,0.882)	**< 0.001**	0.803(0.682,0.945)	**0.008**
CDAI	0.976(0.961,0.991)	**0.001**	0.976(0.961,0.990)	**0.001**	0.983(0.968,0.998)	**0.027**

Model 1: no covariates were adjusted

Model 2: adjusted for gender, age, and race

Model 3: adjusted for gender, age, race, education level, family PIR, BMI, activity condition, and smoking

Abbreviations: *BMI* body mass index; *Family PIR* family poverty income ratio; *CDAI* composite dietary antioxidant index

### The association between antioxidant components and low back pain

We conducted a further analysis on the association between the six antioxidant components of CDAI and low back pain. As shown in Table 4, vitamin E was found to be associated with low back pain in Model 2 (OR = 0.991; 95% CI [0.982, 0.999], *P* = 0.027). After adjusting for all variables, zinc (OR = 1.009; 95% CI [1.002, 1.016], *P* = 0.015) and selenium (OR = 0.379; 95% CI [0.164, 0.875], *P* = 0.023) were independently associated with low back pain. To further explore the non-linear dose-response relationships between antioxidant components and low back pain, we constructed the RCS for six antioxidant components (vitamins A, C, E, carotenes, zinc, and selenium) and low back pain in Model 3. The RCS showed a non-linear dose-response relationship between carotene (P for non-linear = 0.001, L-shaped curve), Zinc (P for non-linear = 0.006, V-shaped curve) Selenium (P for non-linear = 0.034, L-shaped curve) levels and the prevalence of low back pain, respectively (See Supplementary material 2: Figure S2).

**Table 4:** The weighted logistic regression analysis of the association between antioxidant components and low back pain

Components	Model 1		Model 2		Model 3	
	OR [95%CI]	*P*	OR [95%CI]	*P*	OR [95%CI]	*P*
Vitamins A (mg)	1.025(1.000,1.050)	0.055	1.020(0.995,1.045)	0.121	1.022 (0.997,1.048)	0.090
Vitamins C (mg)	1.000(0.999,1.000)	0.103	1.000(0.999,1.000)	0.288	1.000(1.000, 1.001)	0.982
Vitamins E (mg)	0.993(0.985,1.001)	0.091	0.991(0.982,0.999)	**0.027**	0.992(0.984, 1.001)	0.078
Carotene (mg)	0.997(0.993,1.001)	0.105	0.996(0.992,1.000)	0.068	0.997(0.993, 1.001)	0.168
Zinc (mg)	1.008(1.001,1.015)	**0.018**	1.009(1.002,1.016)	**0.009**	1.009(1.002, 1.016)	**0.015**
Selenium (mg)	0.258(0.114,0.586)	**0.001**	0.432(0.188,0.991)	**0.048**	0.379(0.164, 0.875)	**0.023**

Model 1: no covariates were adjusted

Model 2: adjusted for gender, age, and race

Model 3: adjusted for gender, age, race, education level, family PIR, BMI, activity condition, and smoking

*BMI* body mass index; *Family PIR* family poverty income ratio; *CDAI* composite dietary antioxidant index

## Discussion

In our study, the fully adjusted model showed no significant association between CDAI and low back pain. However, subgroup analysis by gender revealed a negative association between CDAI and low back pain in the female population. Females in the highest quartile of CDAI were 19.7% lower likely to experience low back pain compared to those in the lowest quartile. After adjusting for all confounders, we identified zinc and selenium might be independent components associated with low back pain. A dose-response analysis demonstrated a linear association between CDAI and low back pain.

To the best of our knowledge, our study was the first cross-sectional survey to examine the association between CDAI levels and low back pain. Previous researches on dietary interventions for musculoskeletal disorders mostly focused on rheumatoid arthritis, osteoarthritis and fibromyalgia, with relatively limited evidence of low back pain [24–26]. However, accumulating evidence supports oxidative stress as a significant risk factor for low back pain. The inhibition of oxidative stress could maintain redox homeostasis, thereby alleviating low back pain [6]. Consequently, the dietary antioxidant capacity holds great potential in predicting health outcomes in adults [27]. Several studies have explored the association between antioxidants and low back pain. A prospective study suggested that oral antioxidants treatment improves functionality and reduces the use of analgesics in low back pain patients [28]. Results from NHANES also suggested an association between suboptimal vitamin C status and spinal pain [29]. Thus, our study had the potential to further strengthened the link between antioxidants intake and low back pain. Although our study revealed that this negative association was similar in the highest quartile of CDAI, no association between CDAI and low back pain was observed in the fully adjusted model. The controversy may partially stem from the threshold effect of CDAI.

Gender difference plays a significant role in the balance of oxidants [18]. Despite the growing evidence linking antioxidants to low back pain, the impact of gender on outcomes remains ambiguous. A study found that the diet-induced inflammation can affect the experiences of chronic low back pain, with gender significantly modifying the severity of movement-evoked pain [30]. A Czech Republic study reached a similar conclusion that the protective effect of CDAI only appears in women but not in men. This finding suggests that antioxidant properties may prevent disease progression in a gender-specific manner [31]. Gender accounted for the largest proportion of variability in all oxidative stress parameters, with female being more susceptible to oxidative stress [32]. As a result, our study provided further support for this point.

In recent years, there has been growing interest in antioxidants monomers due to their protective roles against oxidative stress-mediated pathological processes [33]. In preclinical experimental studies, numerous individual components with antioxidant activity have been investigated for their potential role in intervertebral disc degeneration, including naringin, salvianolic acid B, quercetin, mangiferin, melatonin, lycopene, and vitamin D [34–40]. In clinical researches, carotenoids and vitamin E have been found to inhibit the formation of lumbar osteophytes in elderly Japanese [41]. Vitamin D supplementation for patients with low back pain can increase serum concentration and reduce oxidative stress in skeletal muscles, leading to a beneficial impact on pain intensity [42]. Our further analysis of antioxidant components revealed selenium was negatively correlated with low back pain, while zinc showed a positive association. Nevertheless, zinc is an important component of antioxidant mitochondrial metalloenzymes, and it can also exert antioxidant effects by binding to metallothioneins [43]. On the other hand, some studies have also expressed the contradictory results. A prospective cohort study in Singapore found that CDAI was beneficial in reducing the risk of colorectal cancer, but did not found any significant association between individual antioxidants and colorectal cancer [44]. Other studies conducted by NHANES have also raised similar concern. The dietary pattern approach recognizes that foods consists of various nutrients and components, that are consumed in combinations and may interact in complex ways [45]. The associations between single components and diseases may be difficult to explore and explain [11]. In addition, we did not collect information about the use of manganese supplements, which differs from some NHANES studies that utilize CDAI. Previous studies have primarily focused on individual antioxidants, but there is a current trend is to increasingly recognize the importance of diet as a whole [46]. Therefore, caution should be exercised when explaining the effects of individual antioxidant components on diseases.

One of the notable strengths of our study is that it was based on NHANES data, which were collected using a stratified multistage probability sampling strategy, making the study more reliable and representative. Furthermore, to the best of our knowledge, this is the first study to investigate the association between CDAI and low back pain. Additionally, we adjusted for confounding factors including gender, age, race, education level, family PIR, BMI, activity condition, and smoking to lessen the impact of confounding. However, this study does have several limitations. Firstly, recall-based questionnaire assessment might involve measurement errors and inaccuracies in assessing antioxidant components. Secondly, bias is inevitable in cross-sectional studies. Moreover, even though we adjusted for some potential confounders, the effect of other potential confounders cannot be completely ruled out.

In conclusion, we observed an inverse association between CDAI and the prevalence of low back pain, with gender differences influencing this association. It is recommended that the antioxidants should be studied in the dietary pattern, and caution should be taken when interpreting the effects of individual antioxidant ingredients. Moreover, considering that diet is a modifiable intervention that has a direct impact on health, further exploration in this area is warranted, especially larger prospective cohort studies.

### Electronic supplementary material

Below is the link to the electronic supplementary material.

Supplementary Material 1

Supplementary Material 2

## Data Availability

The dataset supporting the conclusions of this article is available in the NHANES repository, https://www.cdc.gov/nchs/nhanes.
