# Supplementary material for: Association between different composite dietary antioxidant indexes and low back pain in American women adults: a cross-sectional study from NHANES
Source: BMC Public Health. 2024 Jan 10;24:147. doi: 10.1186/s12889-024-17649-0 (PMC10782773; doi:10.1186/s12889-024-17649-0)
Supplement: Supplementary file 1 — Supplementary Material 1 [file 12889_2024_17649_MOESM1_ESM.docx]

**RCS code**

m1 <- read.csv(“C:/Users/10210/Desktop/Nhanes/Data/lowbackpain.csv”,sep = “,”,header = TRUE)

library(rcssci)

rcssci_linear(knot=4,data=m1, y = (“OUTCOME”),x = “CDAI”,covs=c(“GENDER”, “AGE”, “RACE”, “EDUCATION LEVEL”,“FAMILYPIR”, “BMI”, “ACTIVITYCONDITION”, “SMOKEING”),prob=0.1, filepath=“C:/Users/10210/Desktop/Nhanes/Data”)


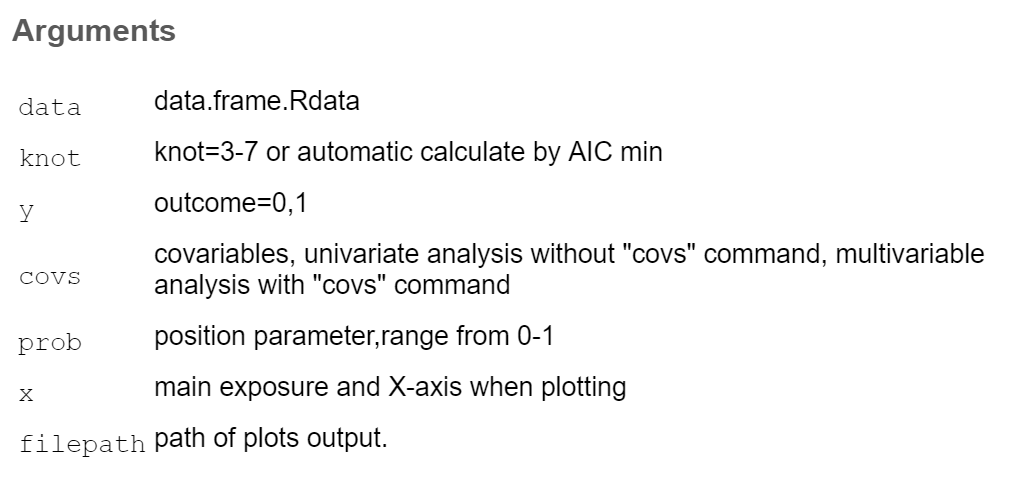


**Logistic code**

demand:

logistic LOWBACKPAIN CDAI ib(1).GENDER AGE ib(1).RACE ib(1).EDUCATION LEVEL ib(1).FAMILYPIR ib(1).BMI ib(1).ACTIVITYCONDITION ib(1).SMOKEING [weight =WEIGHTMEC]
