# Supplementary material for: Association between different composite dietary antioxidant indexes and low back pain in American women adults: a cross-sectional study from NHANES
Source: BMC Public Health. 2024 Jan 10;24:147. doi: 10.1186/s12889-024-17649-0 (PMC10782773; doi:10.1186/s12889-024-17649-0)
Supplement: Supplementary file 2 — Supplementary Material 2 [file 12889_2024_17649_MOESM2_ESM.docx]

**Table S1: The VIF of the covariables in Model 3.**

| Variable | VIF |
| --- | --- |
| Education level | 1.11 |
| Activity condition | 1.10 |
| Gender | 1.09 |
| CDAI | 1.07 |
| Age | 1.06 |
| Smoking | 1.05 |
| Race | 1.05 |
| BMI | 1.04 |
| Family PIR | 1.01 |
| Mean VIF | 1.06 |


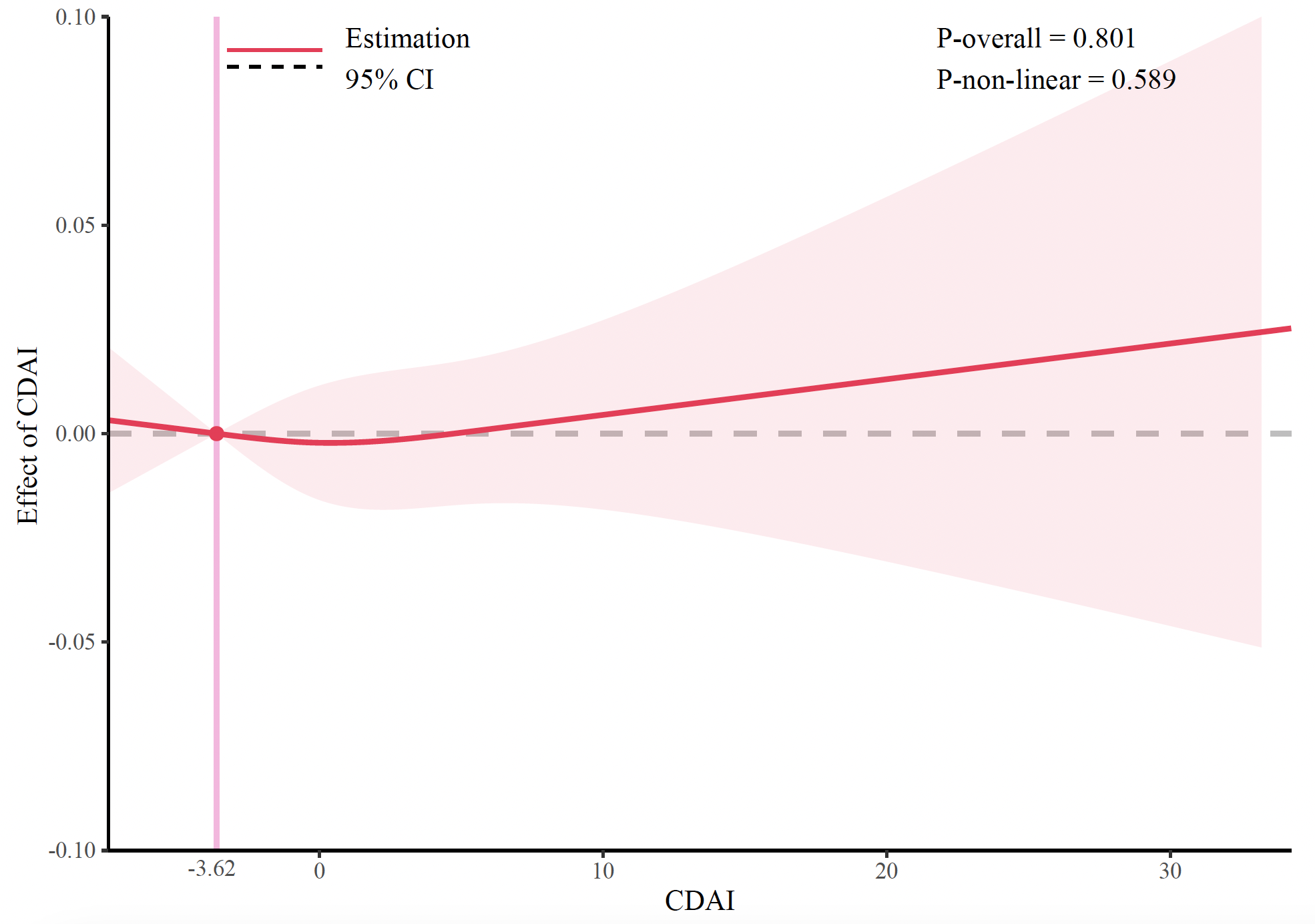


Knot=3


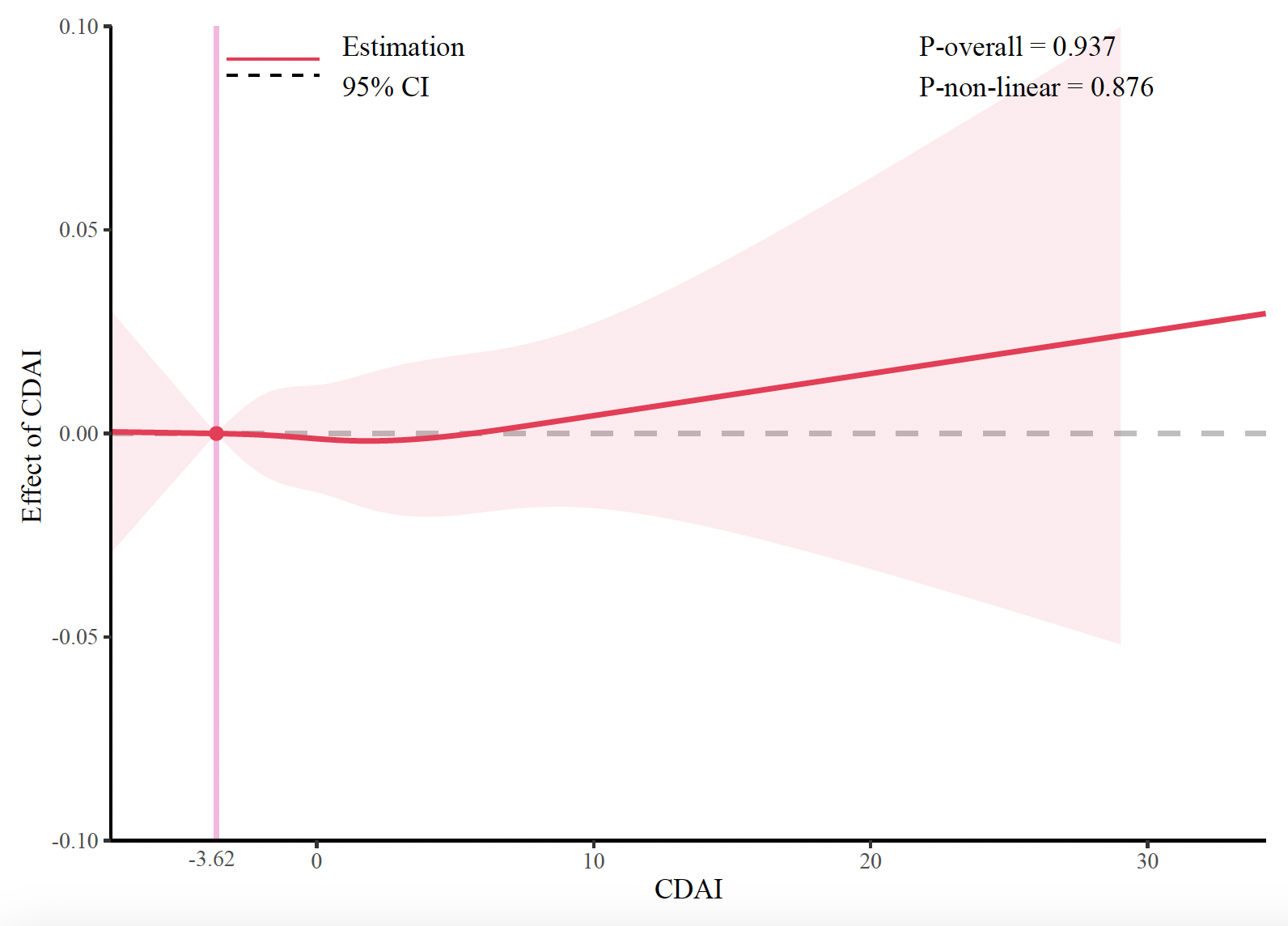

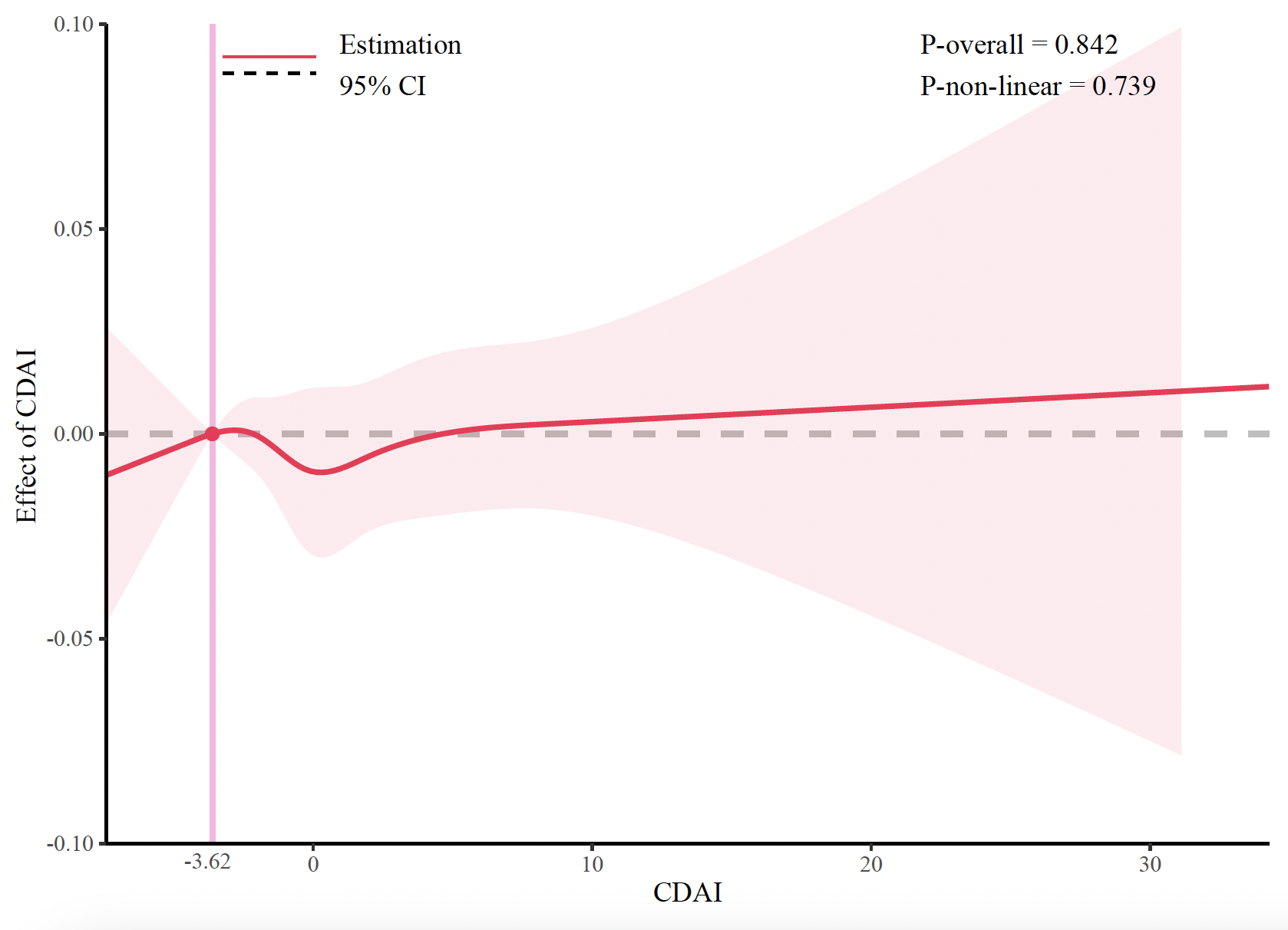


Knot=4 Knot=5


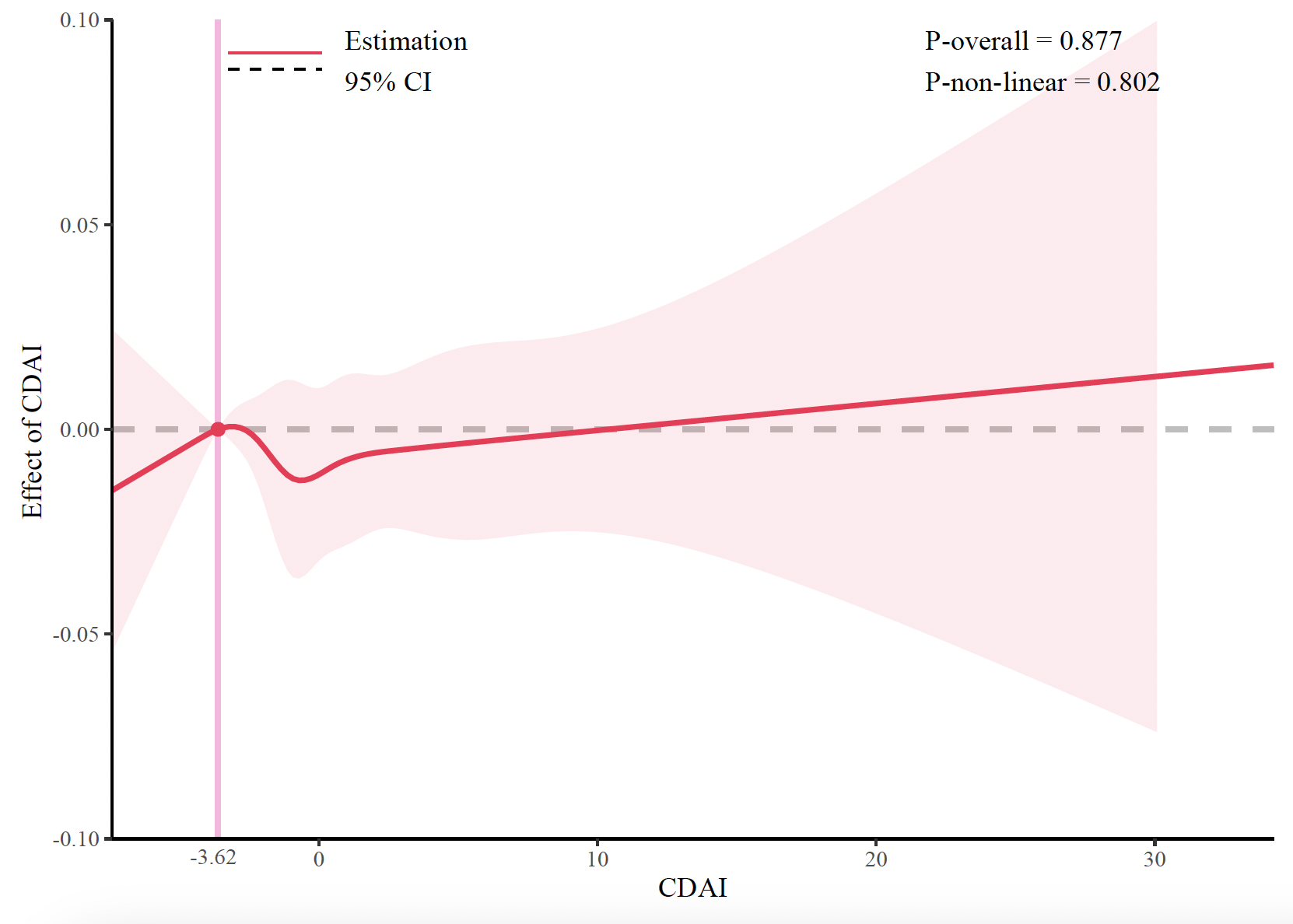

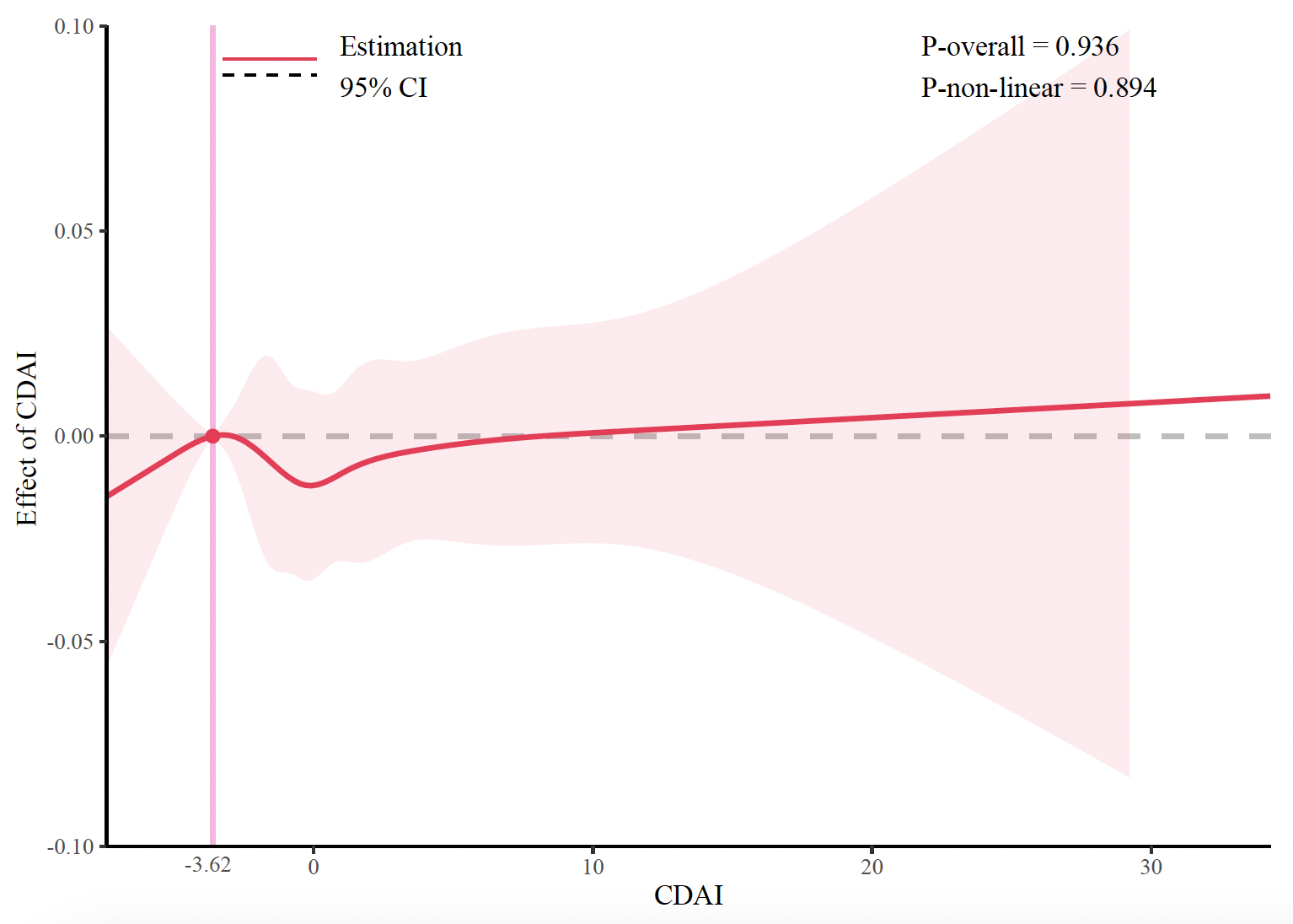


Knot=6 Knot=7

**Figure S1: The dose–response relationships of CDAI with the prevalence of low back pain.**


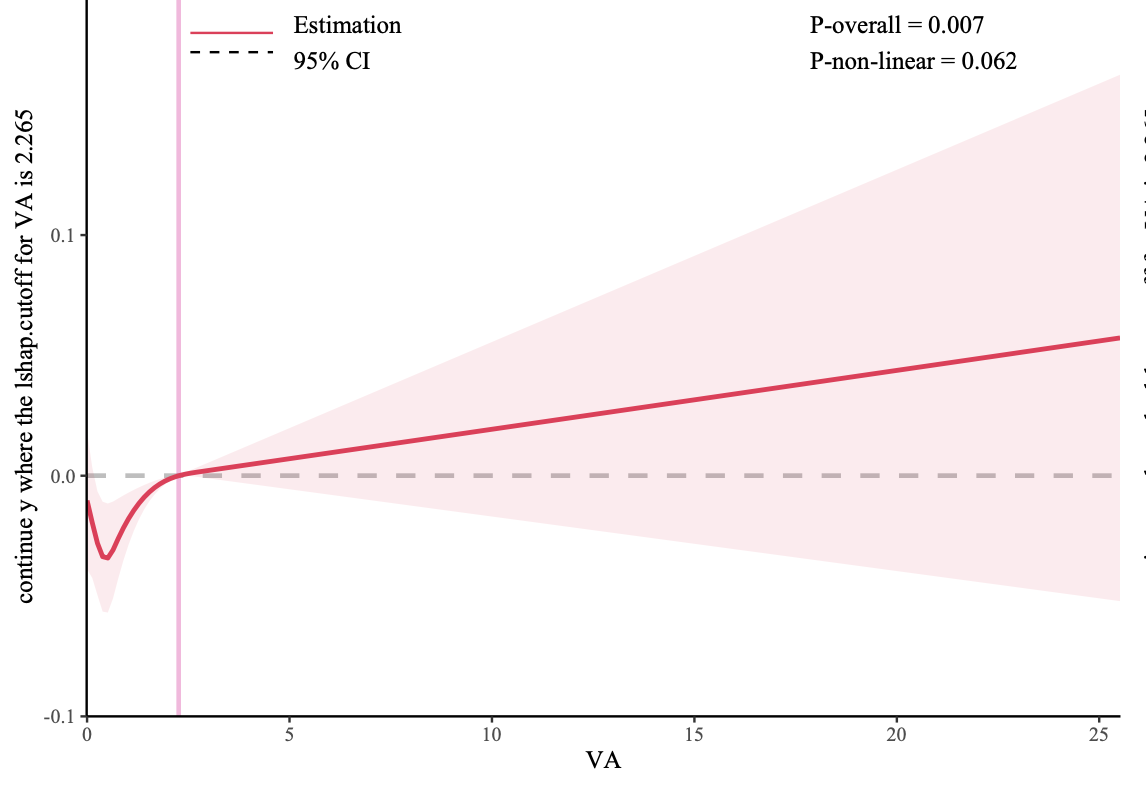

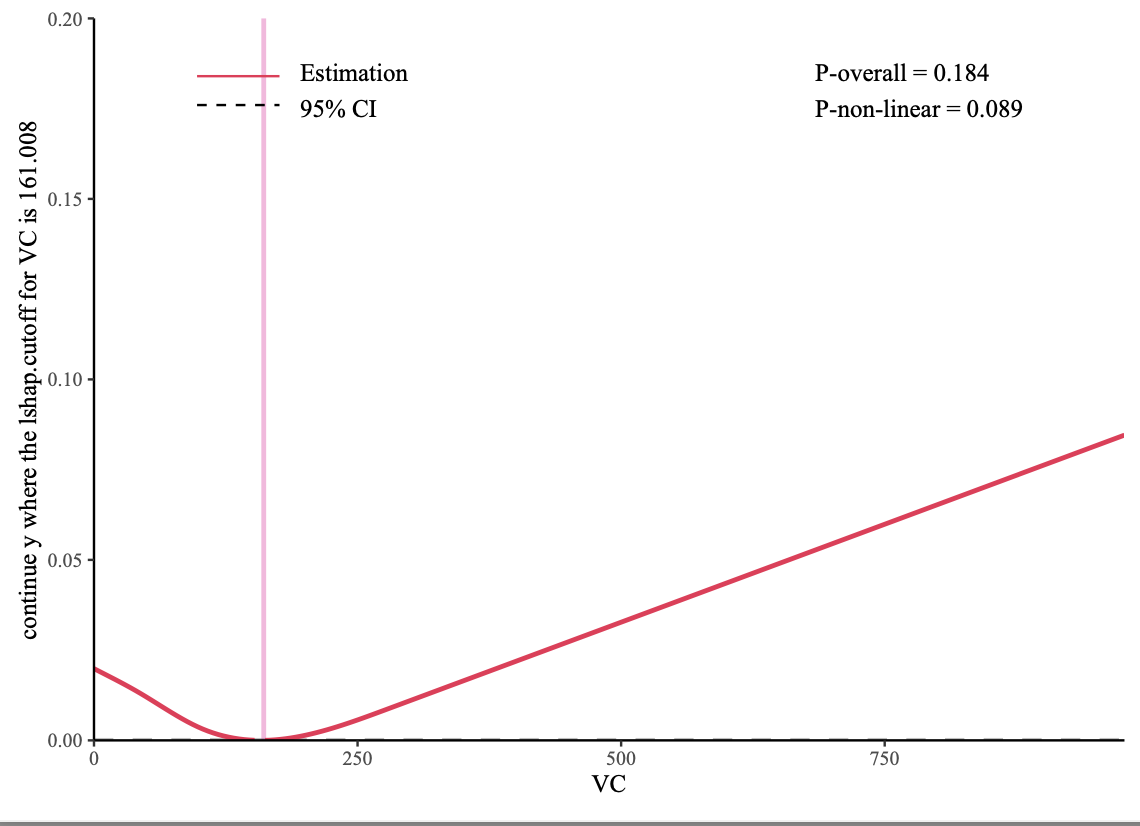

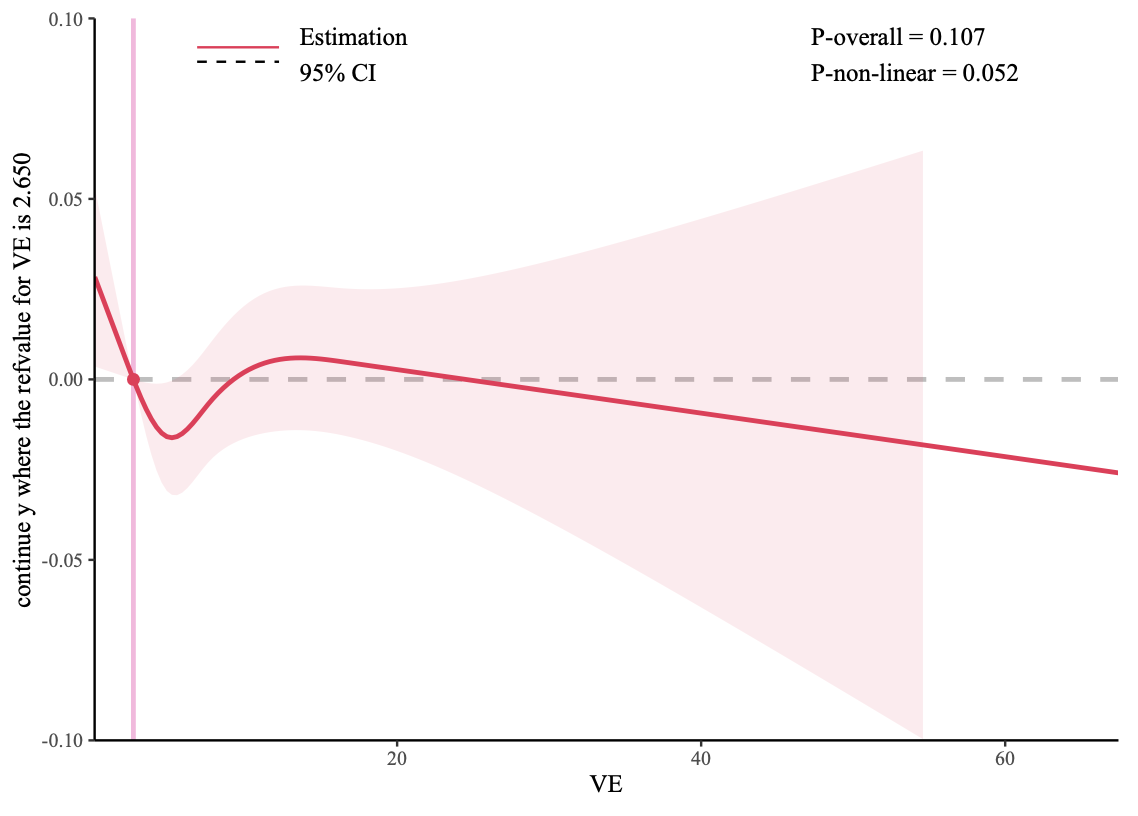

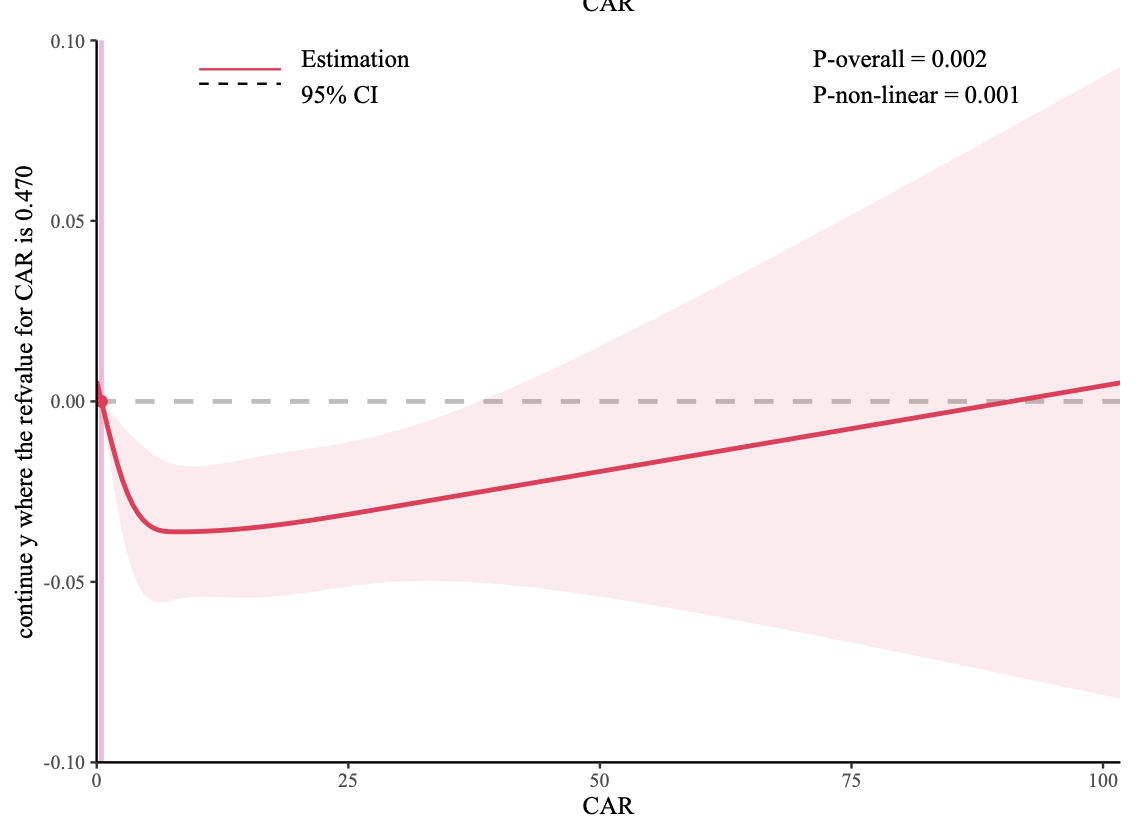

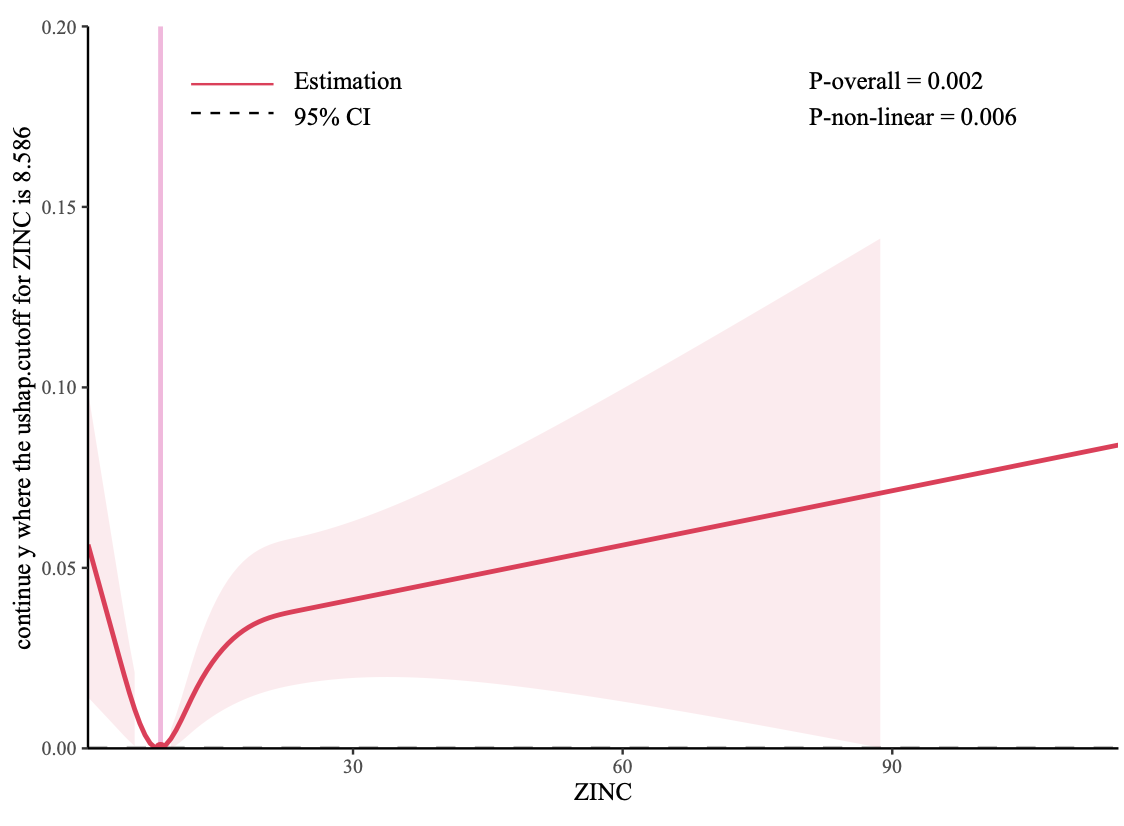

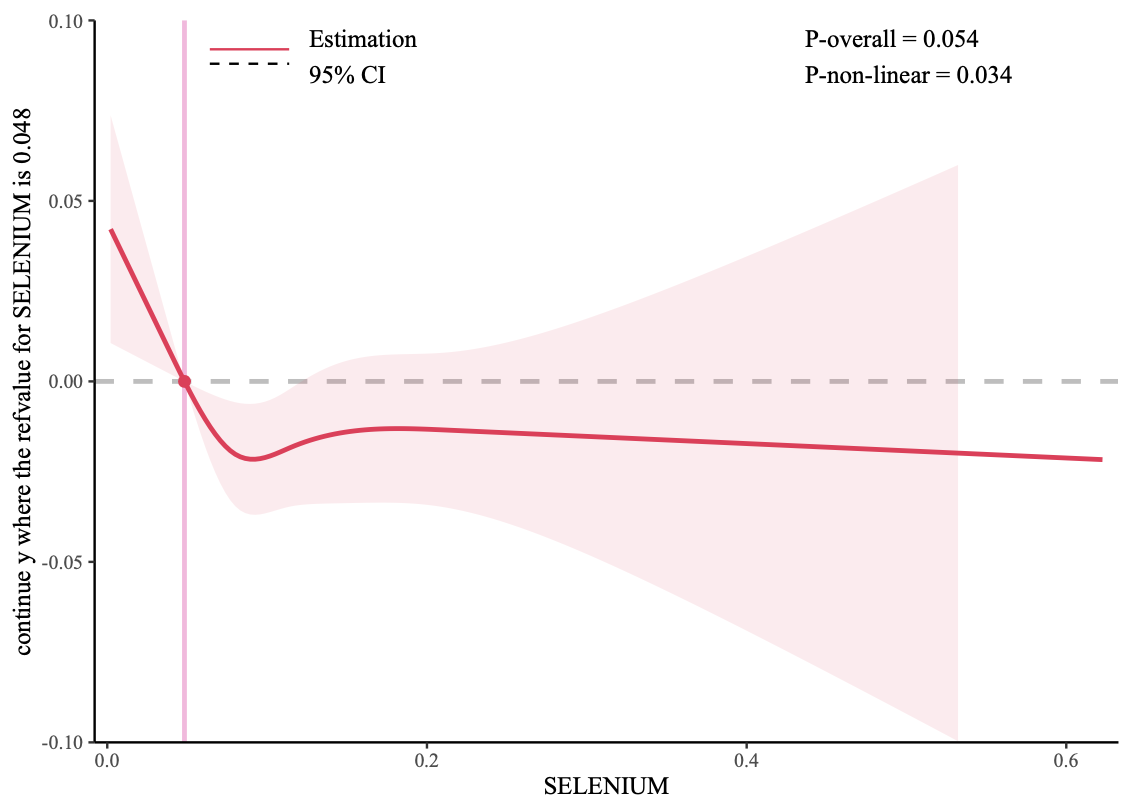


**Figure S2: The dose–response relationships of antioxidant components with the prevalence of low back pain.**
